# A multinational cross-sectional study on the prevalence and predictors of long COVID across 33 countries

**DOI:** 10.1038/s41598-025-10120-z

**Published:** 2025-08-03

**Authors:** Mohamed Sayed Zaazouee, Eman Ayman Nada, Mohammed Al-kafarna, Ahmed Shaheen, Shivabalan Kathavarayan Ramu, Abdelrahman H. Hafez, Sajeda Ghassan Matar, Ahmed Assar, Mohamed Elshennawy, Hazem Abu El-Enien, Hala Jamal Redwan, Sarah Makram Elsayed, Maha Jabir Omran, Omar Hammam Salloum, Hossam Almadhoon, Mohamed Mamdouh, Engy A. Wahsh, Anas Zakarya Nourelden, Alaa Ahmed Elshanbary, Walid Abdel-Aziz, Hivan Haji Rashid, Iman Basheti, Khaled Mohamed Ragab, Ahmed Taher Masoud, Abdelrahman I. Abushouk, Long-COVID International Team (LCIT), Aya Abdalhady Saleh, Ashraf Ahmed Hassan Hamed, Asmaa Gomaa Alwarraqi, Ahmed Mohamed Nour El-deen, Bashar Khaled Almaghary, Hedaya Ramzi Khrawat, Yousef Raid AbdAlrazeq, Saba Usama Turk, Shomoa Alaa Elhajjar, Yousuf Mohammed Deeb, Malak Yousef Badawi, Roa’a Mohammad Aljuneidi, Saja Emad Abusabha, Fida Hussien Al-Ali, Anwar Yousef Jabari, Nusaiba Mohamad Al Zaalan, Ahmed Hashem Fathallah, Dina Osama Yehia Ibrahim, Eman Sayed Mahmoud, Bethel Abraha Gebremeskel, Abdelrahman Mohamed Mahmoud, Mai Mohammad Abdalraouf, Doaa Salah Elgendy, Mostafa Ahmed Elsayed, Hadir Atef Soliman, Manal Mohamed Ali, Mona Abd Elhameed, Asmaa Mohamed Saber, Taghreed Reda Moustafa, Shereen Elsayed Tawfeek, Rania Saad Ramadan, Mahmoud M. Elhady, Arun Quadros Darian, Victor Carrera Gomes, Gabriela Maria Roncon, Fabio Deltreggia, Flavio Castagna de Sousa, João Vitor Eugenio Seabra, Ricardo Silveira Filho, Nada Mohammed Abdel-Aziz Mohammed Zaho, Talha Hannan Chaudhry, Kimanthi Cyrus, Nafisa Agil Hussein, Harshil Girish Patel, Waseth Joy Noela, Preetesh Jakharia, Misbah Shakeel Akhtar, Mohamed Abdelhamid Masoud, Mohamed Saad Elsaied, Mohamed Reda Ezz, Aya Mohamed Rabie Abd Elghany, Ghadeer Magdy Elshafey, Khaled Abouelmagd, Ahmed Gamal Ahmed Shetia, Ghader Mofed Alibrahem, Rasha Hazzaa Ibrahim, Hala Aldeeb, Nouha Halim Moustafa, Alia Mhd Mosalam Al Hosni, Souliman Ahmad Radeef, Lina Dalati, Elaf Sa’ad Aldeen Alashkar, AbdelKader Nabeel Malih, Hisham Alhosni, Mohammed Amir Rais, Bouchra Belhocine, Hadil Elbatoul Meguellatni, Samah Chaib, Hanane Hammoumi, Hadjer Belmerabet, Rayhana Khellafi, Mohammed Zakaria Amrani, Bouthaina Memmou, Abdelkader Kherbache, Jalal Almeer, Salmah Meteran Alrshedi, Amnah A. Alharbi, Adel Mohamed Aboregela, Wessam Abd El Razek Ibrahim Mady, Zenat Khired, Suleiman Ayalew Belay, Elaf Mohamed Hashim Abdelraheem, Tarteel Abdelaal Hassan Omer, Rowa sirelkhatim mohamedahmed, Roaa Ahmed Elobeid, Hamid Magzoub, Fatima Elzahra Ebnomer Mohamed Ibrahim, Basel Adel Mohammed Basrawi, Sarah Fuad Abdo Thabit, Walaa Ismat Mubark Abdelmgeed, Hajar Abdalrazak Alhakari, Fatima Abd Elmonim Mohammed Abd Elkareem, Iman Hisham Abd Elraheem Merghani, Faisal Ismail, Fatma Ahmed Alsharif, Fatima Asedeq Abdulali, Israa Mohammed Benismail, Aisha Hasan Ibreerah, Raneem Ahmed Egzait, Fatima Ibrahim Abushaala, Raga A. Elzahaf, Mahmoud Alfituri Abushiba, Khaled Ali Almehdawi, Haya Ahmed Hijazi, Esraa Emad Alazq, Anas Ishqair, Zakaria Abdallah Abu Elkishik, Taima Nemir Awamleh, Lana Adel Tannous, Nour Mohammad Abumurra, Rayyan Ahmad Al-Qaryouti, Baha’ Ghandi Mohammad Aljeradat, Mai Mahmoud AlAdwan, Rafy Souheil Odeh, Wasan Fawzi Alsafadi, Sally S. Alabdullah, Aseel Ahmad Alkhraibat, Darine Touka, Huzan Hashim Arsalan, Dini Setyowati, Nastiti Faradilla Ramadhani, Alexander Patera Nugraha, Rini Devijanti Ridwan, Diah Savitri Ernawati, Andra Rizqiawan, Ratri Maya Sitalaksmi, Taufan Bramantoro, Beta Novia Rizky, Agung Sosiawan, Tamara Tango, Evangelia Samara, Agathi Karakosta, Pantazi Danai, Gloria Evdoxia Izountouemoi, Konstantinos Stamatis, Ghulam Mujtaba Mughal, Ali Asghar Bhutto, Sidra Absar, Sana Hilal, Anusheh Khan, Sumair Liaqat, Faiza Qamar, Ikram Uddin panhwar, Aayza Ahmed, Javeria Ikram, Syeda Kashfia Haider Zaidi, Sana Asghar, Hafiz Ameer Hamza, Naomi Chinyere Chikezie, Maryam Abdulkarim, Durojaye Aishat Bisoye, Yahaya Fatihu Adam, Jesutomini Esther Abikoye, Opara Franklyn Chiemekam, Al-Awal Abdullah Adedeji, Jeremiah Oluwatomi Itodo Daniel, Bello Saifullah Muhammad, Sara Tavakkoli, Jegr Waad Farj, Darya Salih Hussein, Faheema Rafo Khalaf, Hadiya Rafo Khalaf, Mamend Ibraheem, Hussein Kameran Jabbar, Dlnaz Ayoub Abdi, Sefina Gwachha, Saroma Awal, Bishal Dahal Khatri, Vivekanand lekhak, Belal Shohayeb, Hanem Ellethy, Pop Dariana, Miruna Doru, Rus Nicolae-Dochian, Paul Adrian Pop, Maria Adriana Pop, Rigman Darius-Gabriel, Ene Andrei-Iulian, Bogdan Adrian Buta, Raul Petric, Esmail Abdelmonem, Peter Ghattas, Mira A. O. Alabadla, Tabarak Qassim, Moosa Munawar AlHoda, Joanna Eliza Thomas, Kevin Regi, Mahdi Salah Mahdi, Sornali Roy, Waleed Sameer Abdulkarim Mohamed Affoni, Hasan Alauddeen Yousif Mahmood Alomari, Nour El Houda Ben Jouda, Nayanika Ann Cherian, Devika Nair, Akil Vaibhav, Akash Verma, Rishi Bhatele, Md Ariful Haque, Nazifa Tahseen Akhi, Sabrina Sultana Liza, Hasan Shahriar, Shirsendu Nag Saikat, Fahmida Aslam, Anushka Ann Noor Chowdhury, Khandker Saadiqul Quader, Anju Beesetty, Feven Mekonnen Belay, Michael Azeze Negussie, Fitsum Assefa Gemechu, Samuel Mesfin Girma, Eden Haile Hagos, Ebisa Damesa Amensisa, Mazen El Jamal, Sawsane Ahmad Ghaddar, Zainab Musa Ali, Zeinab Hammoud, Solay Farhat, Ali Ismail, Kazi Hassan Murad, Shahin Ahmed, Akash Saha, Abdinoor Mohamed, Fatema ENur Mou, Ajmina Hasan Flabe, Andrés Estrella, Juan Carlos Panchi Jima, Milton Andrés Méndez Guerrero, María José Rengel Chalco, Walter Alexis Encalada Collahuazo, Carlos Padilla Hernandez, Paola Marlid Donoso Estrada, Karla Alejandra Chiliquinga Jácome, Erika Estefanía Rodríguez Prieto, Niyonkuru Fabrice, Mpawenimana Bosco, Maramuke Carine, Crispine Harushimana, Iteka Délissa Carmella, Irankunda Kévin, Nsabimana Bénit, Mfuranzima Japhet, Irakoze Clovis, Zeynep Ceren Zekey, Selay Doğan, Orgwan Zahra, Mustafa Mehmetoğlu, Eman Khaled, Fouziya Neyyar, Carlos Emilio Hernández Mancia, Iris Rebeca Campos Castro, Carlos Andrés Urrea Valencia, Tania Elizabeth Bolaños Rosero, Priscilla Lopez Ahumada, Narjiss Aji, Arwa Aboumedian, Samia Kessab, Musab Bouhajra, Fatima Zahra Aboumedian, Oumaima Kenna, Amal Shukri Ali Abdulqader Al-Akhdhary, Ahmed Sadeq Maknoon, Nagwan Mansoor Yousef, Abdullah Mohamed Alsoufi, Aiman Al-Khadher, Mohamed Onyango

**Affiliations:** 1 Faculty of Medicine, Al-Azhar University, Assiut, Egypt; 2 Faculty of Pharmacy, Tanta University, Tanta, Gharbia Egypt; 3 School of Public Health, Al-Quds University - Abu Dees, Jerusalem, Palestine; 4 Faculty of Medicine, Alexandria University, Alexandria, Egypt; 5 Heart, Vascular, and Thoracic Institute, Cleveland Clinic Foundation, Cleveland, OH USA; 6 Faculty of Medicine, Al-Azhar University, Cairo, Egypt; 7 Faculty of Pharmacy, Applied Science Private University, Amman, Jordan; 8 Faculty of Medicine, Menofia University, Shibin El Kom, Menofia Egypt; 9 PureHealth, Abu Dhabi, United Arab Emirates; 10 Faculty of Pharmacy, Al-Azhar University, Gaza, Palestine; 11 Faculty of Medicine, October 6 University, Giza, Egypt; 12 Sorbonne Université, Faculté de Médecine, Paris, France; 13 Faculty of Medicine, Palestine Polytechnic University, Hebron, Palestine; 14 Institute of Biodiversity, One Health and Veterinary Medicine, University of Glasgow, Glasgow, UK; 15 Faculty of Medicine, Beni-Suef University, Beni-Suef, Egypt; 16 Faculty of Pharmacy, October 6 University, Giza, Egypt; 17 Faculty of Medicine, Aleppo University, Aleppo, Syria; 18 Pharmaceutical Sciences Department, Faculty of Pharmacy, Jadara University, Irbid, Jordan; 19 Faculty of Medicine and Health, School of Pharmacy, The University of Sydney, Sydney, NSW Australia; 20 Faculty of Medicine, Minia University, Minya, Egypt; 21 Faculty of Medicine, Fayoum University, Fayoum, Egypt; 22 Department of Internal Medicine, Yale School of Medicine, New Haven, CT USA; 23 ALERT Hospital, Addis Ababa, Ethiopia; 24 Faculty of Medicine, Menoufia University, Menoufia, Egypt; 25 Faculty of Medicine, Assiut University, Assiut, Egypt; 26 Faculty of Medicine, Zagazig University, Sharkia, Egypt; 27 Faculty of Medicine, Benha University, Qalubiya, Egypt; 28 Faculdade de Medicina, Universidade Municipal de São Caetano do Sul, São Caetano do Sul, SP Brazil; 29 Faculty of Medicine, Suez Canal University, Ismailia, Egypt; 30 Faculty of Medicine, University of Nairobi, Nairobi, Kenya; 31 Faculty of Medicine, Tanta University, Gharbia, Egypt; 32 Public Health and Community Medicine Department, Faculty of Medicine, Tanta University, Tanta, Egypt; 33 Electron Microscope Department, Theodor Bilharz Research Institute, Giza, Egypt; 34 Faculty of Medicine, Al-Azhar University, New Damietta, Egypt; 35 Faculty of Medicine, Ain Shams University, Cairo, Egypt; 36 Faculty of Health, Upo University, Novara, Italy; 37 Taqati Medical Institute, Faculty of Medicine, Tishreen University, Lattakia, Syria; 38 Faculty of Pharmacy, Tishreen University, Latakia, Syria; 39 Faculty of Pharmacy, Tartous University, Tartous, Syria; 40 Faculty of Pharmacy, Arab International University, Ghabaghib, Syria; 41 Faculty of Medicine, Damascus University, Damascus, Syria; 42 Faculty of Pharmacy, Damascus University, Damascus, Syria; 43 Faculty of Medicine, University of Kalamoon, Rif Dimashq Governorate, Deir Atiyah, Syria; 44 Faculty of Medicine, Imperial College London, London, UK; 45 Faculty of Medicine of Algiers, University of Algiers 1, Algiers, Algeria; 46 Faculty of Medicine, Constantine, Algeria; 47 Faculty of Medicine, Abou Bekr Belkaid, Tlemcen, Algeria; 48 Faculty of Medicine, Salah Boubnider University, Constantine, Algeria; 49 Faculty of Medicine, Ahmed Ben Bella University, Oran, Algeria; 50 Faculty of Medicine, Batna University, Batna, Algeria; 51 Telecommunications Engineer, Jaen University, Jaén, Spain; 52 Dr. Haifa Eye Hospital, Manama, Bahrain; 53 Faculty of Nursing, Taibah University, Madinah, Kingdom of Saudi Arabia; 54 Department of Biochemistry, Faculty of Science, University of Tabuk, Tabuk, Kingdom of Saudi Arabia; 55 College of Medicine, University of Bisha, Bisha, Saudi Arabia; 56 King Fahad Medical Research Center, King Abdulaziz University, Jeddah, Kingdom of Saudi Arabia; 57 Jazan University, Jizan, Kingdom of Saudi Arabia; 58 Department of Medicine, University of Gondar, Gondar, Ethiopia; 59 Faculty of Medicine, University of Khartoum, Khartoum, Sudan; 60 Faculty of Medicine, Al-Neelain University, Khartoum, Sudan; 61 Federal Ministry of Health, Khartoum, Sudan; 62 Department Clinical Laboratory, Faculty of Medical Technology, University of Tobruk, Tobruk, Libya; 63 National Centre for Disease Control, Tobruk, Libya; 64 Libyan Medical Research Centre, Kambut, Tobruk, Libya; 65 Faculty of Medical Technology, University of Tripoli, Tripoli, Libya; 66 Faculty of Medicine, Misurata University, Misurata, Libya; 67 Department of Public Health, College of Medical Technology, Derna, Libya; 68 MENA Research Group, Derna, Libya; 69 Faculty of Science, Azzaytuna University, Tarhuna, Libya; 70 Ministry of Health, Benghazi, Libya; 71 Faculty of Medicine, Al Azhar University, Gaza, Palestine; 72 Faculty of Applied Medical Sciences, Al-Azhar University, Gaza, Palestine; 73 Faculty of Medicine, Hashemite University, Zarka, Jordan; 74 Faculty of Medicine, Jordan University, Amman, Jordan; 75 Faculty of Pharmacy, Jordan University of Science and Technology, Irbid, Jordan; 76 Faculty of Pharmacy, Duhok University, Kurdistan, Iraq; 77 Faculty of Dental Medicine, Universitas Airlangga, Surabaya, Indonesia; 78 Faculty of Medicine, Universitas Indonesia, Jakarta, Indonesia; 79 Faculty of Medicine, University of Ioannina, Ioannina, Greece; 80 Evangelismos Hospital, Athens, Greece; 81 Faculty of Pharmacy, University of Sindh, Jamshoro, Sindh Pakistan; 82 College of Pharmacy, Liaquat University of Medical & Health Sciences (LUMHS), Jamshoro, Sindh Pakistan; 83 Faculty of Pharmacy, University of Karachi, Karachi, Pakistan; 84 Punjab University College of Pharmacy, Lahore, Pakistan; 85 Department of Pharmacy, Lahore College Pharmaceutical Sciences, Lahore, Pakistan; 86 Jinnah Sindh Medical University, Karachi, Pakistan; 87 Department of Pharmacy, University of Peshawar, Peshawar, Pakistan; 88 Faculty of Pharmacy, Hamdard University Islamabad, Islamabad, Pakistan; 89 Jr Hospital Pharmacist, Institute of Radiotherapy and Nuclear Medicine’s Patient Welfare Society, Peshawar, KPK Pakistan; 90 Karachi Medical and Dental College, Karachi, Pakistan; 91 Department of Pharmacy, Hamdard University Islamabad Campus, Chak Shahzad, Islamabad Pakistan; 92 Faculty of Pharmaceutical Sciences, University of Port Harcourt, Port Harcourt, Nigeria; 93 Faculty of Health Science, National Open University, Minna Study Center, Niger, Nigeria; 94 Faculty of Pharmaceutical Sciences, University of Ilorin, Ilorin, Nigeria; 95 Faculty of Veterinary Medicine, University of Ilorin, Ilorin, Nigeria; 96 Obafemi Awolowo University, Ile-Ife, Osun State Nigeria; 97 College of Health Science, Bayero University, Kano, Nigeria; 98 Faculty of Pharmacy, Tabriz Medical University, Tabriz, Iran; 99 College of Pharmacy, University of Duhok, Duhok, Kurdistan Region Iraq; 100 Department of Pharmacy, Kathmandu University, Dhulikhel, Kavre Nepal; 101 School of International Education, Hainan Medical University, Xueyuan Road, Longhua District, Haikou, Hainan China; 102 Asian College for Advance Studies, Purbanchal University, Lalitpur, Nepal; 103 Queensland Brain Institute, The University of Queensland, Brisbane, Australia; 104 School of Information Technology and Electrical Engineering, University of Queensland, St Lucia, Australia; 105 George Emil Palade University of Medicine, Pharmacy, Sciences and Technology from Târgu Mureș, Târgu Mureș, Romania; 106 Department of Biology, College of Staten Island, The City University of New York, New York, NY USA; 107 Fairleigh Dickinson University, Morris County, NJ USA; 108 School of Medicine, Royal College of Surgeons in Ireland-Bahrain, Busaiteen, Bahrain; 109 Faculty of Medicine, First Moscow State Medical University named after I.M. Sechenov, Moscow, Russia; 110 Department of Orthopedic Surgery, Yan’an Hospital Affiliated to Kunming Medical University, Kunming, Yunnan China; 111 Biochemistry and Molecular Biology, Tianjin Medical University, Tianjin, China; 112 Kunming Medical University, Yunnan, China; 113 Faculty of Clinical Medicine, Ningxia Medical University, Yinchuan, Ningxia China; 114 International Food and Drug Policy Law and Research Center, Shenyang Pharmaceutical University, Shenyang, China; 115 School of Pharmaceutical Sciences, Tsinghua University, Beijing, China; 116 China Three Gorges University, Yichang, Hubei China; 117 East Kent Hospitals University NHS Foundation Trust - Kent and Canterbury Hospital, Canterbury, Kent England; 118 Soochow University, Suzhou, China; 119 School of Medicine, College of Health Sciences, Addis Ababa University, Addis Ababa, Ethiopia; 120 Faculty of Medicine, Lebanese University, Beirut, Lebanon; 121 Department of Public Health, Atish Dipankar University of Science & Technology, Dhaka, Bangladesh; 122 BDRCS Field Hospital, Bangladesh Red Crescent Society, PMO, Cox’s Bazar, Bangladesh; 123 Emergency Medical Officer, Oasis Hospital, Sylhet, Bangladesh; 124 Sir Salimullah Medical College, Dhaka, Bangladesh; 125 School of Medicine, Kenyatta University, Nairobi, Kenya; 126 Radiology & Imaging, Mugda Medical Collage Hospital, Dhaka, Bangladesh; 127 Clinical Trial Coordinator, BADAS Paediatric Diabetes Centre, Dhaka, Bangladesh; 128 Pontificia Universidad Católica del Ecuador, Quito, Ecuador; 129 Hospital Shell, Shell, Mera Canton, Pastaza Province Ecuador; 130 Facultad de Medicina, Universidad de las Americas, Quito, Ecuador; 131 Universidad Espiritu Santo, Guayaquil, Ecuador; 132 Fundacion Vozandes, Carapungo, Quito Ecuador; 133 CUCS, Universidad de Guadalajara, Guadalajara, Mexico; 134 Universidad Nacional Autónoma de México, Coyoacán, Mexico; 135 Hospital de Alta Especialidad del Bajío, Residente Pediatria, León-Guanajuato, Mexico; 136 Faculty of Health Sciences, Frank Ogden School of Medicine, Hope Africa University, Bujumbura, Burundi; 137 Faculty of Engineering and Technology, Hope Africa University, Bujumbura, Burundi; 138 Faculty of Medicine, Ankara University, Ankara, Turkey; 139 Pharmacist In Charge, Protos Pharmaceuticals, Dubai, United Arab Emirates; 140 Universidad de El Salvador, San Salvador, El Salvador; 141 CENTRO MEDICO CLINIC SALUD ISLA DE MAIPO CHILE, Isla de Maipo, Chile; 142 Faculty of Medicine and Pharmacy of Rabat, Mohamed V University, Rabat, Morocco; 143 Dermatology Resident et al-Thawra Hospital- Arab Board of Health Specialization, Sanaa, Yemen; 144 College of Medicine, University of Hadhramout, Hadhramout, Yemen; 145 London School of Hygiene and Tropical Medicine (LSHTM), University of London, London, UK; 146 Faculty of Medicine, Sana’a University, Sanaa, Yemen; 147 Médecins Sans Frontières (MSF), Aden, Yemen; 148 Thamar University Faculty of Medicine, Thamar, Yemen; 149 Faculty of Health Sciences, University of Nairobi, Nairobi, Kenya

**Keywords:** Covid-19, Long Covid, Multi-national, Re-infection, Vaccination, Infectious diseases, Risk factors

## Abstract

The symptoms of long COVID (LC) can be debilitating and may be associated with anxiety, social stigma, and quality of life deterioration. Identifying patients at risk of LC is important to offer follow-up care and plan population-level public health measures. The current multinational study aimed to assess the prevalence and predictors of LC in the general population. We conducted an online, multinational, cross-sectional survey between April 2022 and January 2023, targeting participants 18 years and older with a previously confirmed COVID-19 infection. We used convenience sampling to recruit participants through an online Google form. We collected demographic data, past medical history, infection details, post-COVID-19 symptoms, and quality of life. Responses were then translated into English. LC was defined as per the World Health Organization. A single-variable analysis was conducted to identify factors significantly associated with LC development. Following the removal of multicollinear variables, a generalized linear model was established to estimate the contribution of different predictors to LC occurrence. A total of 11,801 respondents from 33 countries were included in the analysis. The mean age for participants was 32.7 ± 12.8 years, with 61% being females. BMI averaged 25.2 ± 4.8 across participants, and 14.8% of them were smokers. Seventy-eight percent of participants reported receiving the COVID-19 vaccine. Respondents with PCR-confirmed COVID-19 were then categorized into those with LC (N = 2335, 19.8%) and without LC (N = 9466 individuals, 80.2%). Our model identified 25 significant predictors. The predictors of higher LC risk included ICU admission (OR 2.08; 95% CI 1.36, 3.18; *P* = 0.001), female sex (OR 1.8; 95% CI 1.61, 2.02; *P* < 0.001), fatigue during the infection (OR 1.6; 95% CI 1.43, 1.78; *P* < 0.001), identifying as Hispanic (OR 1.53; 95% CI 1.26, 1.85; *P* < 0.001), and pre-existing gastrointestinal disease (OR 1.48; 95% CI 1.22, 1.8; *P* < 0.001). In conclusion, we identified key LC predictors, including ICU admission, female sex, and acute fatigue as primary risk factors, while African American and Asian ethnicities and receiving even one dose of vaccination demonstrated protective effects.

## Introduction

Post-viral syndromes in the coronavirus family were observed in previous diseases related to coronavirus, such as severe acute respiratory syndrome (SARS) and Middle East respiratory syndrome (MERS)^1–3^. Patients reported experiencing fatigue, myalgia, and psychiatric symptoms for as long as four years, and some individuals who survived SARS were symptomatic for up to 7 and 15 years^4^. A few months into the COVID-19 pandemic, reports of protracted COVID-19 cases began to surface. Initially, healthcare professionals (HCPs) dismissed these concerns, attributing them to anxiety and stress. However, as the frequency of such cases continued to rise, HCPs embarked on a systematic inquiry into the potential enduring consequences of COVID-19^5,6^.

This long-term constellation of symptoms and signs, currently labeled as long COVID syndrome (LC), has become widely recognized among scientific communities. There are various definitions of LC, but the commonly utilized definition includes symptoms persisting beyond three months after the initial onset of the disease^7,8^. LC represents a major public health challenge, affecting approximately 20% of COVID-19 survivors^9^. Previous Literature show 41.7% of patients experience persistent symptoms two years post-infection, with 14.1% unable to return to work^10^. Chronic fatigue syndrome affects 21.4% of laboratory-confirmed cases, creating substantial healthcare system burden globally^11^. Less common symptoms encompass mental and cognitive disorders, headaches, joint and chest pains, hair loss, and cardiac and gastrointestinal issues. These symptoms can last six months or more after symptom onset. LC strikes individuals of all disease severities, including mild cases and younger adults who escaped hospitalization and respiratory support^12^. Even children, including those who had asymptomatic COVID-19, can endure debilitating symptoms for extended periods^13^.

LC pathophysiology is characterized by interconnected mechanisms that collectively contribute to its multisystem clinical manifestations. Viral persistence involves prolonged SARS-CoV-2 detection in multiple tissue compartments^14^, while mitochondrial dysfunction results in impaired cellular bioenergetics and oxidative stress^15^. Concurrently, immune dysregulation manifests through persistent inflammatory responses and autoimmune processes^16^. Additionally, autonomic nervous system dysfunction presents cardiovascular dysautonomia affecting heart rate variability^17^. These mechanisms interact synergistically to produce the complex symptomatology observed in LC patients.

While some studies have attempted to understand the predictors of LC development among COVID-19 patients^18,19^, most of these studies were small-sized and they did not use a standardized approach to defining LC or confirming COVID-19 infection^20^. Further investigation and research are needed to understand the range and variability of LC symptoms fully. In our study, we aim to fill the gap in the literature by assessing the prevalence and predictors of LC symptoms, their association with demographic characteristics, COVID-19 severity, and the quality of life of COVID-19 survivors by conducting a global cross-sectional study. Further, identifying patients at risk of LC is important to offer follow-up care and plan population-level public health measures.

## Methods

### Study settings

From April 2022 to January 2023, we conducted a multinational cross-sectional survey using online questionnaires. Eligibility spanned individuals aged 18 and above in 33 selected countries who had a PCR-confirmed COVID-19 diagnosis. Participants self-reported the timing of their COVID-19 infection and symptom onset, with PCR confirmation required for inclusion. The questionnaire development process is detailed in Supplementary File 1.

### Sampling and data source

We used convenience sampling to recruit participants by distributing the online survey. The sample size of 348 was based on a standard calculation assuming an expected proportion (P) of 50% to maximize sample size with minimal prior assumptions. This was applied per country to ensure representativeness. We did not use different *P* values for each country to maintain consistency. Using 50% is common to provide a conservative estimate. Since we have 43 predictors in our regression model, we used the Events Per Variable (EPV) method to calculate the minimum total sample size. Assuming 20 EPV, a minimum of 860 participants were required to achieve 10 EPV. To adopt a more conservative approach with 20 EPV, a total of 1720 participants was needed from all countries. Collaborators received instructions on the data collection strategy, with a central investigator from each country overseeing the process to ensure balanced participation.

### Data collection and handling

We collected participation details, demographics, medical history, COVID-19 infection course, post-COVID symptoms (neurological/cardiac/respiratory/mental health), and quality of life (physical/emotional/social impacts). Symptom assessment focused on those persisting at the time of survey completion. Participants were specifically asked to report symptoms that occurred after COVID-19 infection and persisted for > 12 weeks, distinguishing them from acute illness symptom. Participants were explicitly asked about PCR confirmation status and symptom duration. In addition, data were obtained on vaccination status, treatments, and long-term effects. Data was gathered through Google Forms and shared via social media (Facebook, Twitter, WhatsApp, and LinkedIn) with repeated postings. Each participant was allowed a single survey response to prevent duplicates. In addition, we reviewed the timestamps manually for any inconsistencies. Responses from each country were automatically translated into English and then reviewed by a bilingual translator. Finally, we compiled all data into a single datasheet for analysis.

### Primary outcome

The primary outcome of interest included identifying predictors LC in PCR-positive COVID-19 patients. Long Covid syndrome was defined as “Development of signs and symptoms during or after an infection consistent with findings typical of COVID‑19 that continue for more than 12 weeks and cannot be matched to an alternative diagnosis”^21,22^. This operational definition ensured capture of participants with symptoms consistent with LC, distinct from acute infection symptoms.

### Statistical analysis

We used the Kolmogorov–Smirnov Z test to assess normality. Descriptive statistics summarized percentages for categorical variables, and both means (± standard deviation [SD]) and medians with interquartile ranges [IQR] for continuous data. Correlations between continuous variables and LC development were evaluated using point biserial coefficients to identify important variables for subsequent analysis. The Chi-Square test examined associations between the outcome variable and categorical variables. For logistic regression, we tested the linearity of continuous variables using the Box-Tidewell test. Then we conducted a univariate logistic regression for reference. To account for intervariable confounding in the multivariate model, a preliminary logistic regression model was built to predict LC development, followed by multicollinearity assessment with variance inflation factors (VIFs). Only one variable from each set of collinear variables was retained in the final model. The model performance was assessed with Tjur’s R^2^ and Akaike information criterion (AIC). Analyses were conducted using R (version 4.3.2; R Project for Statistical Computing), with statistical significance set at a two-tailed *P* value < 0.05.

### Ethical considerations

Our study protocol was approved by the Institutional Review Board of the Faculty of Medicine, Tanta University, Gharbia, Egypt (Approval number: 35960/10/22) and by the faculty of pharmacy, Applied science Private University, Amman, Jordan (Approval number: 2022-PHA-30). Informed consent was obtained from all study participants at the start of the online questionnaire after explaining the goal and methods of study. No personal data were collected. The study with conducted adhering to the tenets of the Declaration of Helsinki.

## Results

### Population characteristics

A total of 26,125 Participants responded to our questionnaire. Of them, 1,127 were excluded due to incomplete or invalid responses. Of the remaining 24,998 participants with reported COVID-19 infection, we included 11,801 participants who had a PCR-confirmed COVID-19 infection. According to the prespecified criteria, these patients were then annotated as those with (N = 2335, 19.8%) or without LC (N = 9466, 80.2%). In the total sample, 7196 (61%) were males, the mean age was 31.4 ± 8.5 years. The most frequently observed symptoms of Long COVID were chest pain (n = 610, 30.0%), shortness of breath (n = 898, 27.1%), dysgeusia (n = 849, 25.5%), insomnia (n = 254, 26.7%), muscle/joint symptoms (n = 1603, 24.4%), fatigue (n = 1622, 24.2%), and gastrointestinal symptoms (n = 1129, 23.3%). Detailed countries and participant numbers are presented in Supplementary Table 1, and the baseline characteristics are illustrated in Tables 1 and 2. Univariate estimates for different predictors are available in Supplementary Table 2.

**Table 1:** Demographic and clinical characteristics of 11,801 COVID-19 patients, stratified by long-COVID status.

Label	Variable	Long-COVID		Total (N: 11,801)	Difference (*P* value)
		No (N: 9466)	Yes (N: 2335)		
Age (Years)	Min/Max	18.0/88.0	18.0/88.0	18.0/88.0	0.040
	Med [IQR]	28.0 [23.0;40.0]	29.0 [23.0;41.0]	28.0 [23.0;40.0]	
	Mean (std)	32.6 (12.7)	33.3 (13.3)	32.7 (12.8)	
Sex	Female	5468 (75.9%)	1728 (24.0%)	7196 (60.9%)	≤ 0.001
	Male	3998 (86.8%)	607 (13.1%)	4605 (39.0%)	
Ethnicity	Asian	1862 (87.8%)	257 (12.1%)	2119 (17.9%)	≤ 0.001
	Black or African American	793 (89.8%)	90 (10.1%)	883 (7.4%)	
	Hispanic or Latino	546 (72.0%)	212 (27.9%)	758 (6.4%)	
	Indian	490 (85.9%)	80 (14.0%)	570 (4.8%)	
	Middle Eastern	2315 (78.1%)	647 (21.8%)	2962 (25.1%)	
	White/Caucasian	2758 (75.8%)	879 (24.1%)	3637 (30.8%)	
	Other	702 (80.5%)	170 (19.5%)	872 (7.3%)	
BMI (Kg/m^2^)	Min/Max	12.6/50.0	13.7/47.9	12.6/50.0	0.034
	Med [IQR]	24.5 [21.8;27.7]	24.8 [22.0;28.0]	24.6 [21.8;27.8]	
	Mean (std)	25.1 (4.8)	25.4 (5.0)	25.2 (4.8)	
Smoking	Ex-smoker	481 (81.2%)	111 (18.7%)	592 (5.0%)	0.001
	No	7526 (79.5%)	1933 (20.4%)	9459 (80.1%)	
	Yes	1459 (83.3%)	291 (16.6%)	1750 (14.8%)	
Number of cigarettes (Cigarettes)	Min/Max	0/40.0	0/40.0	0/40.0	0.001
	Med [IQR]	0 [0;0]	0 [0;0]	0 [0;0]	
	Mean (std)	1.8 (5.3)	1.6 (5.2)	1.8 (5.3)	
Smoking duration (Years)	Min/Max	0/5.0	0/5.0	0/5.0	0.002
	Med [IQR]	0 [0;0]	0 [0;0]	0 [0;0]	
	Mean (std)	0.6 (1.4)	0.6 (1.4)	0.6 (1.4)	
COVID vaccine received	Not Vaccinated	1879 (72.8%)	701 (27.1%)	2580 (21.8%)	≤ 0.001
	Vaccinated	7587 (82.2%)	1634 (17.7%)	9221 (78.1%)	
Vaccine Doses	0	1197 (70. 83%)	493 (29.1%)	1690 (14.3%)	≤ 0.001
	1	524 (80.2%)	129 (19.7%)	653 (5.5%)	
	2	222 (90.2%)	24 (9.7%)	246 (2.0%)	
	4	7523 (81. 67%)	1689 (18.3%)	9212 (78.0%)	
Isolation	Home	8164 (79.8%)	2061 (20.1%)	10,225 (86.6%)	≤ 0.001
	ICU	173 (69.7%)	75 (30.2%)	248 (2.1%)	
	Inpatient	771 (84.9%)	137 (15.0%)	908 (7.6%)	
	No	358 (85.2%)	62 (14.7%)	420 (3.5%)	
Reinfection	No	7602 (81.1%)	1762 (18.8%)	9364 (79.3%)	≤ 0.001
	Yes	1864 (76.4%)	573 (23.5%)	2437 (20.6%)	

Min, Minimum; Max, Maximum; Med, Median; IQR, Interquartile Range; std, Standard Deviation; N, Number; BMI, Body Mass Index.

**Table 2:** Prevalence of acute and chronic symptoms among COVID-19 patients with and without long-COVID.

Label	Variable	Long-COVID		Total (N: 11,801)	Difference (*P* value)
		No (N: 9466)	Yes (N: 2335)		
Respiratory parameters					
Anosmia	No	7290 (79.9%)	1832 (20.0%)	9122 (77.3%)	0.135
	Yes	2176 (81.2%)	503 (18.7%)	2679 (22.7%)	
	Total	9466 (80.2%)	2335 (19.7%)	11,801 (100.0%)	
Asthma	No	8732 (80.3%)	2137 (19.6%)	10,869 (92.1%)	0.244
	Yes	734 (78.7%)	198 (21.2%)	932 (7.9%)	
	Total	9466 (80.2%)	2335 (19.7%)	11,801 (100.0%)	
Chest pain	No	8047 (82.3%)	1725 (17.6%)	9772 (82.8%)	≤ 0.001
	Yes	1419 (69.9%)	610 (30.0%)	2029 (17.1%)	
	Total	9466 (80.2%)	2335 (19.7%)	11,801 (100.0%)	
Cough	No	4062 (79.8%)	1023 (20.1%)	5085 (43.0%)	0.431
	Yes	5404 (80.4%)	1312 (19.5%)	6716 (56.9%)	
	Total	9466 (80.2%)	2335 (19.7%)	11,801 (100.0%)	
Runny nose	No	6286 (79.8%)	1587 (20.1%)	7873 (66.7%)	0.152
	Yes	3180 (80.9%)	748 (19.0%)	3928 (33.2%)	
	Total	9466 (80.2%)	2335 (19.7%)	11,801 (100.0%)	
SOB	No	7053 (83.0%)	1437 (16.9%)	8490 (71.9%)	≤ 0.001
	Yes	2413 (72.8%)	898 (27.1%)	3311 (28.0%)	
	Total	9466 (80.2%)	2335 (19.7%)	11,801 (100.0%)	
Sore throat	No	5750 (81.0%)	1349 (19.0%)	7099 (60.1%)	0.009
	Yes	3716 (79.0%)	986 (20.9%)	4702 (39.8%)	
	Total	9466 (80.2%)	2335 (19.7%)	11,801 (100.0%)	
Constitutional and neurologic					
Confusion	No	8659 (80.3%)	2112 (19.6%)	10,771 (91.2%)	0.116
	Yes	807 (78.3%)	223 (21.6%)	1030 (8.7%)	
	Total	9466 (80.2%)	2335 (19.7%)	11,801 (100.0%)	
Conjunctivitis	No	9003 (80.0%)	2238 (19.9%)	11,241 (95.2%)	0.134
	Yes	463 (82.6%)	97 (17.3%)	560 (4.7%)	
	Total	9466 (80.2%)	2335 (19.7%)	11,801 (100.0%)	
Dysgeusia	No	6987 (82.4%)	1486 (17.5%)	8473 (71.8%)	≤ 0.001
	Yes	2479 (74.4%)	849 (25.5%)	3328 (28.2%)	
	Total	9466 (80.2%)	2335 (19.7%)	11,801 (100.0%)	
Fatigue	No	4404 (86.0%)	713 (13.9%)	5117 (43.3%)	≤ 0.001
	Yes	5062 (75.7%)	1622 (24.2%)	6684 (56.6%)	
	Total	9466 (80.2%)	2335 (19.7%)	11,801 (100.0%)	
Fever	No	4138 (80.9%)	977 (19.1%)	5115 (43.3%)	0.102
	Yes	5328 (79.6%)	1358 (20.3%)	6686 (56.6%)	
	Total	9466 (80.2%)	2335 (19.7%)	11,801 (100.0%)	
History of stroke	No	9370 (80.2%)	2312 (19.7%)	11,682 (98.9%)	0.900
	Yes	96 (80.6%)	23 (19.3%)	119 (1.0%)	
	Total	9466 (80.2%)	2335 (19.7%)	11,801 (100.0%)	
Migraine	No	8481 (81.2%)	1954 (18.7%)	10,435 (88.4%)	≤ 0.001
	Yes	985 (72.1%)	381 (27.8%)	1366 (11.5%)	
	Total	9466 (80.2%)	2335 (19.7%)	11,801 (100.0%)	
Muscle joint symptoms	No	4499 (86.0%)	732 (13.9%)	5231 (44.3%)	≤ 0.001
	Yes	4967 (75.6%)	1603 (24.4%)	6570 (55.6%)	
	Total	9466 (80.2%)	2335 (19.7%)	11,801 (100.0%)	
Rash	No	8956 (80.1%)	2224 (19.8%)	11,180 (94.7%)	0.219
	Yes	510 (82.1%)	111 (17.8%)	621 (5.2%)	
	Total	9466 (80.2%)	2335 (19.7%)	11,801 (100.0%)	
GI symptoms	No	5758 (82.6%)	1206 (17.3%)	6964 (59.0%)	≤ 0.001
	Yes	3708 (76.6%)	1129 (23.3%)	4837 (40.9%)	
	Total	9466 (80.2%)	2335 (19.7%)	11,801 (100.0%)	
Insomnia	No	8772 (80.8%)	2081 (19.1%)	10,853 (91.9%)	≤ 0.001
	Yes	694 (73.2%)	254 (26.7%)	948 (8.0%)	
	Total	9466 (80.2%)	2335 (19.7%)	11,801 (100.0%)	
Comorbidities					
Anemia	No	8751 (80.8%)	2072 (19.1%)	10,823 (91.7%)	≤ 0.001
	Yes	715 (73.1%)	263 (26.8%)	978 (8.2%)	
	Total	9466 (80.2%)	2335 (19.7%)	11,801 (100.0%)	
Autoimmune disease	No	9079 (80.4%)	2202 (19.5%)	11,281 (95.5%)	0.001
	Yes	387 (74.4%)	133 (25.5%)	520 (4.4%)	
	Total	9466 (80.2%)	2335 (19.7%)	11,801 (100.0%)	
Cancer	No	9402 (80.2%)	2314 (19.7%)	11,716 (99.2%)	0.253
	Yes	64 (75.2%)	21 (24.7%)	85 (0.7%)	
	Total	9466 (80.2%)	2335 (19.7%)	11,801 (100.0%)	
COPD	No	9364 (80.2%)	2303 (19.7%)	11,667 (98.8%)	0.232
	Yes	102 (76.1%)	32 (23.8%)	134 (1.1%)	
	Total	9466 (80.2%)	2335 (19.7%)	11,801 (100.0%)	
Diabetes type 1	No	9293 (80.1%)	2304 (19.8%)	11,597 (98.2%)	0.097
	Yes	173 (84.8%)	31 (15.2%)	204 (1.7%)	
	Total	9466 (80.2%)	2335 (19.7%)	11,801 (100.0%)	
Diabetes type 2	No	9106 (80.2%)	2243 (19.7%)	11,349 (96.1%)	0.757
	Yes	360 (79.6%)	92 (20.3%)	452 (3.8%)	
	Total	9466 (80.2%)	2335 (19.7%)	11,801 (100.0%)	
Immunosuppression	No	9239 (80.3%)	2263 (19.6%)	11,502 (97.4%)	0.059
	Yes	227 (75.9%)	72 (24.0%)	299 (2.5%)	
	Total	9466 (80.2%)	2335 (19.7%)	11,801 (100.0%)	
GI disease	No	9051 (80.8%)	2146 (19.1%)	11,197 (94.8%)	≤ 0.001
	Yes	415 (68.7%)	189 (31.2%)	604 (5.1%)	
	Total	9466 (80.2%)	2335 (19.7%)	11,801 (100.0%)	
Heart disease	No	9173 (80.1%)	2266 (19.8%)	11,439 (96.9%)	0.725
	Yes	293 (80.9%)	69 (19.0%)	362 (3.0%)	
	Total	9466 (80.2%)	2335 (19.7%)	11,801 (100.0%)	
Hypertension	No	8652 (80.3%)	2121 (19.6%)	10,773 (91.2%)	0.385
	Yes	814 (79.1%)	214 (20.8%)	1028 (8.7%)	
	Total	9466 (80.2%)	2335 (19.7%)	11,801 (100.0%)	
Renal disease	No	9362 (80.2%)	2304 (19.7%)	11,666 (98.8%)	0.352
	Yes	104 (77.0%)	31 (22.9%)	135 (1.1%)	
	Total	9466 (80.2%)	2335 (19.7%)	11,801 (100.0%)	
Seizure	No	9357 (80.2%)	2310 (19.8%)	11,667 (98.8%)	0.741
	Yes	109 (81.3%)	25 (18.6%)	134 (1.1%)	
	Total	9466 (80.2%)	2335 (19.7%)	11,801 (100.0%)	

Min, Minimum; Max, Maximum; Med, Median; IQR, Interquartile Range; std, Standard Deviation; N, Number; SOB, Shortness of Breath; GI, Gastrointestinal; COPD, Chronic Obstructive Pulmonary Disease.

### Exploratory data analysis

Ethnicity, number of vaccine doses, and smoking were negatively correlated with the outcome; however, all correlations were weak (Table 3). Seventeen categorical variables, mostly dichotomous, significantly contributed to the development of LC during the single-variable analysis Tables 1 and 2. The five predictors with the highest odds were the presence of chest pain (OR 2.01; 95% CI 1.80, 2.23), both malaise and muscle symptoms (OR 1.98; 95% CI 1.80, 2.18), pre-existing GI disease (OR 1.92; 95% CI 1.60, 2.29), and shortness of breath (OR 1.83; 95% CI 1.66, 2.01). We excluded the number of vaccine doses from further analysis due to multicollinearity.

**Table 3:** Correlation coefficients between demographic/clinical factors and long-COVID development.

Parameter	r	95% CI	*P* value
Age	0.02	(0.00, 0.04)	0.151
BMI	0.02	(0.00, 0.04)	0.151
Number of Cigarettes	− 0.02	(− 0.03, 0.00)	0.556
Ethnicity	− 0.10	(− 0.12, − 0.08)	< 0.001
Vaccine doses	− 0.08	(− 0.1, − 0.06)	< 0.001
Smoking duration	− 0.02	(− 0.03, 0.00)	0.614
Smoking	− 0.03	(− 0.04, − 0.01)	0.047
Isolation	0.01	(0.00, 0.03)	0.614

R, Correlation coefficient; CI, Confidence Interval.

### Predictors of Long Covid

The multivariable logistic regression model revealed 25 significant predictors of Long COVID, with adjusted odds ratios (AOR) as follows. Significant predictors of a higher risk of developing LC in order of effect size magnitude were ICU admission (AOR 2.08; 95% C.I. 1.36, 3.18; *P* = 0.001), female sex (AOR 1.8; 95% C.I. 1.61, 2.02; *P* < 0.001), fatigue during the infection (AOR 1.6; 95% C.I. 1.43, 1.78; *P* < 0.001), identifying as Hispanic (AOR 1.53; 95% C.I. 1.26, 1.85; *P* < 0.001), pre-existing gastrointestinal disease (AOR 1.48; 95% C.I. 1.22, 1.8; *P* < 0.001), muscle and joint pain (AOR 1.44; 95% C.I. 1.29, 1.61; *P* < 0.001), developing shortness of breath (AOR 1.43; 95% C.I. 1.28, 1.6; *P* < 0.001), dysgeusia (AOR 1.41; 95% C.I. 1.26, 1.59; *P* < 0.001), chest pain (AOR 1.33; 95% C.I. 1.17, 1.51; *P* < 0.001), being diagnosed with migraine (AOR 1.3; 95% C.I. 1.13, 1.49; *P* < 0.001), re-infection with COVID-19 (AOR 1.2; 95% C.I. 1.07, 1.34; *P* = 0.002) and older age (AOR 1.008; 95% C.I. 1.004, 1.012; *P* < 0.001).

On the other hand, the following factors were associated with a lower risk of developing LC: identifying as African American (AOR 0.37; 95% C.I. 0.29, 0.47; *P* < 0.001) or Asian (AOR 0.5; 95% C.I. 0.42, 0.59; *P* < 0.001), receiving COVID-19 vaccine (AOR 0.62; 95% C.I. 0.54, 0.71; *P* < 0.001), developing conjunctivitis (AOR 0.68; 95% C.I. 0.53, 0.87; *P* = 0.002), having Indian origin (AOR 0.68; 95% C.I. 0.52, 0.88; *P* = 0.004), being a current smoker (AOR 0.71; 95% C.I. 0.53, 0.95; *P* = 0.022), pre-existing heart disease (AOR 0.73; 95% C.I. 0.54, 0.98; *P* = 0.041) or anemia (AOR 0.77; 95% C.I. 0.63, 0.93; *P* = 0.008), and developing rash (AOR 0.77; 95% C.I. 0.61, 0.97; *P* = 0.028), runny nose (AOR 0.81; 95% C.I. 0.73, 0.91; *P* < 0.001), cough (AOR 0.85; 95% C.I. 0.77, 0.95; *P* = 0.003), or fever (AOR 0.86; 95% C.I. 0.77, 0.96; *P* = 0.005); Table. It is noteworthy that the coefficient of determination of the model was 0.08, indicating high variability which could not be accounted for by the linear model (Table 4).

**Table 4:** Multivariable logistic regression analysis of long-COVID risk factors: adjusted odds ratios for demographic, clinical, and symptom-based predictors.

Parameter	(OR 95% CI; *P* value)
Age	1.01 (1, 1.01; *P* ≤ 0.001)
Female sex	1.80 (1.61, 2.02; *P* ≤ 0.001)
BMI	1.01 (1, 1.02; *P* = 0.128)
Ethnicity	
White/Caucasian	Ref
Hispanic or Latino	1.53 (1.26, 1.85; *P* ≤ 0.001)
Middle eastern	0.94 (0.83, 1.06; *P* = 0.3)
Black or African American	0.37 (0.29, 0.47; *P* ≤ 0.001)
Asian	0.50 (0.42, 0.59; *P* ≤ 0.001)
Indian	0.68 (0.52, 0.88; *P* = 0.004)
Other	0.77 (0.63, 0.93; *P* = 0.008)
Smoking	
Non-Smoker	Ref
Current Smoker	0.71 (0.53, 0.95; *P* = 0.022)
Ex-smoker	0.93 (0.7, 1.22; *P* = 0.619)
Smoking duration (Years)	1.06 (0.99, 1.15; *P* = 0.11)
Number of cigarettes	1.00 (0.99, 1.01; *P* = 0.996)
Respiratory parameters	
Anosmia	0.92 (0.8, 1.05; *P* = 0.214)
Asthma	1.01 (0.84, 1.2; *P* = 0.942)
Chest pain	1.33 (1.17, 1.51; *P* ≤ 0.001)
Cough	0.85 (0.77, 0.95; *P* = 0.003)
Runny nose	0.81 (0.73, 0.91; *P* ≤ 0.001)
SOB	1.43 (1.28, 1.6; *P* ≤ 0.001)
Sore throat	1.02 (0.92, 1.14; *P* = 0.659)
Constitutional and neurologic	
Confusion	1.03 (0.86, 1.23; *P* = 0.748)
Conjunctivitis	0.68 (0.53, 0.87; *P* = 0.002)
Dysgeusia	1.41 (1.26, 1.59; *P* ≤ 0.001)
Fatigue	1.60 (1.43, 1.78; *P* ≤ 0.001)
Fever	0.86 (0.77, 0.96; *P* = 0.005)
History stroke	0.82 (0.48, 1.35; *P* = 0.446)
Migraine	1.30 (1.13, 1.49; *P* ≤ 0.001)
Muscle joint	1.44 (1.29, 1.61; *P* ≤ 0.001)
Rash	0.77 (0.61, 0.97; *P* = 0.028)
GI symptoms	1.04 (0.94, 1.16; *P* = 0.424)
Insomnia	1.15 (0.97, 1.35; *P* = 0.116)
Comorbidities	
Anemia	0.77 (0.63, 0.93; *P* = 0.008)
Autoimmune disease	1.04 (0.84, 1.3; *P* = 0.693)
Cancer	1.35 (0.78, 2.24; *P* = 0.266)
COPD	1.30 (0.81, 2.04; *P* = 0.268)
Type 1 diabetes	0.75 (0.49, 1.13; *P* = 0.181)
Type 2 diabetes	0.87 (0.66, 1.14; *P* = 0.318)
Immunosuppression	0.86 (0.64, 1.15; *P* = 0.325)
GI disease	1.48 (1.22, 1.8; *P* ≤ 0.001)
Heart disease	0.73 (0.54, 0.98; *P* = 0.041)
Hypertension	0.92 (0.76, 1.1; *P* = 0.358)
Renal disease	1.19 (0.75, 1.85; *P* = 0.439)
Seizure	0.89 (0.53, 1.43; *P* = 0.634)
Isolation	
No Isolation	Ref
Home isolation	1.22 (0.92, 1.65; *P* = 0.171)
Hospital isolation in floor	1.08 (0.76, 1.53; *P* = 0.672)
Hospital isolation in ICU department	2.08 (1.36, 3.18; *P* = 0.001)
Reinfection	1.20 (1.07, 1.34; *P* = 0.002)
Received one or more doses of COVID vaccine	0.62 (0.54, 0.71; *P* ≤ 0.001)

OR, Odds Ratio; CI, Confidence Interval; BMI, Body Mass Index; SOB, Shortness of Breath; GI, Gastrointestinal; COPD, Chronic Obstructive Pulmonary Disease; Ref, Reference variable.

## Discussion

This multicenter study of 11,801 PCR-confirmed COVID-19 patients (19.8% developed Long COVID) identified 25 significant predictors through multivariable logistic regression analysis. The strongest risk factors for Long COVID development were ICU admission, female sex, acute-phase fatigue, Hispanic ethnicity, pre-existing gastrointestinal disease, and acute symptoms including muscle/joint pain, shortness of breath, dysgeusia, and chest pain. Conversely, protective factors included African American and Asian ethnicity, COVID-19 vaccination, conjunctivitis, Indian origin, current smoking, and, unexpectedly, pre-existing heart disease and anemia, along with certain acute symptoms like cough and fever. These findings emphasize the heterogeneous nature of Long COVID and highlight the need for personalized risk-stratification approaches to better understand the underlying mechanisms driving symptom persistence in COVID-19 survivors.

Moreno-Pérez and colleagues investigated the prevalence and factors associated with LC. Their study found that LC was detected in approximately 50.9% of patients, with varying incidence rates based on COVID severity. Notably, patients with severe pneumonia had a cumulative incidence of 58.2%, while those with mild pneumonia and without pneumonia had 36.6% and 37.0%, respectively. Unlike our findings, age, sex, comorbidities, and acute COVID-19 severity were independent predictors of LC. However, lung opacities > 50% and higher heart rates at admission in severe pneumonia cases were identified as independent predictors of LC^23^.

The extant literature exhibits variability regarding the correlation between the female gender and the presence of LC. Some preliminary investigations have indicated an elevated occurrence of fatigue and other symptoms in women^24,25^, while other studies have not identified a gender association^26–28^. Discrepancies in ethnicity, geographical location, and socio-economic status could account for these divergent findings. Hormones might contribute to the perpetuation of the hyperinflammatory state experienced during the acute phase even after convalescence^29,30^, and there is evidence of heightened production of IgG antibodies in females during the early stages of the disease, which could potentially lead to more favorable outcomes for women^31^. However, this also may play a role in the persistence of disease manifestations.

Similar to our findings, previous literature showed that advanced age was associated with persistent fatigue, musculoskeletal discomfort, and impairment in pulmonary capabilities, mirroring a decrement in organ performance and a comparatively gradual recuperative capacity^25^. Also, Baratta et al. corroborated the link between disease severity and enduring symptoms^32^. Nevertheless, instances of LC have previously been documented among individuals not admitted to hospitals diagnosed with a mild and self-remitting ailment^23–25^.

Budhiraja et al. observed that approximately 15% of individuals in their study reported symptoms persisting for over 4 weeks, with 11% experiencing symptoms for more than one year. Distinct dissimilarities were evident in the particular symptoms, with certain symptoms more prevalent among vaccinated individuals and others more frequent among the unvaccinated cohort. However, the comprehensive impact of vaccination, including the vaccine type administered, did not significantly influence the prevalence or duration of LC^33^. Viet-Thi et al. revealed a correlation between the amelioration of LC symptoms and the administration of vaccinations^34^. According to the hypothesis advanced to elucidate these observations, a theory previously assumed by Molnar et al., the ailment’s progression is potentially driven by a persisting viral reservoir and/or circulating viral fragments. The COVID-19 vaccine could potentially stimulate and activate the entire immune system, eradicating these persisting viral antigens and consequent enhancement of symptomatology^35^. An additional survey encompassing 900 LC patients, conducted by a UK-based advocacy group, reported that 56.7% of participants noticed an improvement in their symptoms following their initial COVID-19 vaccine dose. However, the precise underlying mechanism remains to be definitively ascertained^36^.

The protective association of COVID-19 vaccination may be explained by the vaccine’s ability to reduce viral load and duration of infection, potentially limiting tissue damage and subsequent inflammatory cascades^37^. The inverse relationship between certain acute symptoms (conjunctivitis, rash, runny nose, cough, and fever) and LC risk might reflect different immunological responses or viral tropism patterns that paradoxically result in more effective viral clearance^38^. The protective effect observed in current smokers is counterintuitive but has been noted in previous studies and might relate to nicotine’s immunomodulatory effects on ACE2 expression or cytokine responses^39,40^. Similarly, the protective associations with pre-existing heart disease and anemia could suggest altered baseline inflammatory states or medication effects that inadvertently modify the post-infection immune response^41^. These findings underscore the complex pathophysiology of LC and highlight the need for mechanistic studies to understand these protective relationships.

Our analysis identified multiple predictors for LC development that included both demographic and disease-specific criteria e.g. clinical manifestations, severity, and re-infection. Interestingly, receiving even one dose of the COVID-19 vaccination was associated with lower odds of developing LC. Of course. Here is a revised and consolidated paragraph that integrates all the limitations into a formal, scientific format. However, our study was not free of limitations that warrant consideration. First, from a statistical standpoint, our logistic regression model yielded a low pseudo-R^2^ value (0.08) and a high AIC (10,776). While this preserves the interpretation of predictor direction and relative importance, the low R^2^ limits the interpretability of the absolute magnitude of the effect sizes, and the high AIC suggests considerable model complexity that may indicate non-linear relationships in the data. Second, the study’s methodological design has inherent constraints. The cross-sectional nature precludes causal inference, and our reliance on a self-report online questionnaire introduces potential for self-selection and recall bias. Although eligibility required self-reported PCR confirmation, these results could not be independently verified. Our distribution method via social media also prevented the calculation of a formal response rate, and the exclusion of incomplete responses may have introduced sampling bias. Finally, the ethnicity-based analyses were not adjusted for country of residence, which may have allowed for residual confounding. Future research is needed to corroborate these findings, potentially with longitudinal designs, and to identify additional predictors that better explain the variance in LC occurrence.

## Clinical implications and future directions

The identification of key predictors for LC has critical clinical relevance. Risk stratification based on factors such as ICU admission, gender, acute-phase symptoms, and pre-existing conditions can help clinicians proactively monitor high-risk patients and initiate early supportive interventions. Conversely, recognition of protective factors, such as vaccination, certain ethnic backgrounds, and specific acute symptoms, may inform public health strategies and patient counseling. The observed protective effect of even a single vaccine dose reinforces the need for continued vaccination advocacy, particularly in populations with lower uptake.

Future research should prioritize prospective, longitudinal studies incorporating objective biomarkers, imaging, and validated clinical assessments to overcome the limitations of self-reported, cross-sectional data. Mechanistic studies are essential to elucidate the immunological, virological, and genetic underpinnings of both risk and protective factors, particularly in understanding paradoxical findings such as the protective association with smoking and pre-existing heart disease. Furthermore, predictive models incorporating non-linear dynamics and multi-omic data should be developed to enhance individualized care pathways. Ultimately, a precision medicine approach integrating demographic, clinical, and immunologic profiles is needed to inform targeted therapies and rehabilitation strategies for LC patients.

## Conclusion

Our large-scale analysis identified key LC predictors, with ICU admission, female sex, and acute fatigue as primary risk factors, while African American and Asian ethnicities and receiving even one dose of the vaccination showed protective effects. Future research should employ longitudinal designs with objective clinical measures, explore additional biological and psychosocial predictors, and investigate non-linear relationships to better understand long COVID pathogenesis. Priority should be given to developing more robust predictive models that can guide clinical decision-making and targeted interventions for high-risk populations.

## Supplementary Information

Supplementary Information 1.

Supplementary Information 2.

## Data Availability

The datasets generated during and/or analyzed during the current study are available from the corresponding author on reasonable request.
