# Supplementary material for: A multinational cross-sectional study on the prevalence and predictors of long COVID across 33 countries
Source: Sci Rep. 2025 Aug 3;15:28299. doi: 10.1038/s41598-025-10120-z (PMC12319108; doi:10.1038/s41598-025-10120-z)
Supplement: Supplementary file 1 — Supplementary Information 1. [file 41598_2025_10120_MOESM1_ESM.docx]

**Questionnaire Development**

The questionnaire was developed based on a comprehensive review of existing literature on post-COVID-19 symptoms, guidelines from the World Health Organization (WHO), and Centers for Disease Control and Prevention (CDC), as well as previously published surveys on long-term COVID-19 effects. The draft was reviewed by department heads and external specialists to ensure face validity, relevance, comprehensiveness, and clarity of each section.

The final questionnaire included the following sections:

► Invitation to Participate: Provided information about the purpose of the study, inclusion criteria, duration, risks, benefits, and confidentiality.

► Demographic Data: Collected participants’ age, gender, country of residence, living area (urban/suburban/rural), ancestry, education level, employment status, and basic health information (e.g., smoking status, blood type).

► Past Medical History: Assessed pre-existing conditions (e.g., autoimmune diseases, cardiovascular diseases, respiratory diseases, metabolic syndromes) and medication/supplement use before COVID-19 infection. Participants were also asked about influenza vaccination history and COVID-19 vaccination status.

► COVID-19 Infection Details: Included questions about the type of COVID-19 diagnosis (confirmed, suspected, or asymptomatic), duration of infection, symptoms during the acute phase, oxygen saturation levels, and medical interventions (e.g., oxygen therapy, mechanical ventilation).

► Post-COVID-19 Symptoms: Focused on long-term symptoms experienced after recovery from COVID-19, such as neurological issues (e.g., brain fog, memory attenuation), cardiac symptoms, respiratory issues, and mental health impacts. Participants rated the severity and duration of these symptoms.

► Quality of Life Assessment: Evaluated participant's self-reported health status, physical limitations, pain interference, and emotional well-being (e.g., calmness, energy levels, feelings of depression) over the past four weeks. Social activity limitations due to physical or emotional problems were also assessed.

The questionnaire was pilot-tested with a small group of participants to ensure clarity and ease of completion. Based on feedback, minor adjustments were made to question phrasing and formatting. The final version was translated into multiple languages using a forward-backward translation method to ensure cultural and linguistic appropriateness. Internal consistency of each section was assessed using Cronbach’s alpha for a subset of initial responses (not included in the final analysis).
