# Supplementary material for: A multinational cross-sectional study on the prevalence and predictors of long COVID across 33 countries
Source: Sci Rep. 2025 Aug 3;15:28299. doi: 10.1038/s41598-025-10120-z (PMC12319108; doi:10.1038/s41598-025-10120-z)
Supplement: Supplementary file 2 — Supplementary Information 2. [file 41598_2025_10120_MOESM2_ESM.docx]

Table S1. Geographic Distribution of Participant Responses to Our Survey.

| Country | Total Responses | Confirmed PCR | | |
| --- | --- | --- | --- | --- |
|  |  | Long Covid | No Long Covid | Total |
| Algeria | 1877 | 134 (17.1%) | 649 (82.9%) | 783 |
| Australia | 375 | 51 (27.7%) | 133 (72.3%) | 184 |
| Bahrain | 503 | 65 (15.8%) | 346 (84.2%) | 411 |
| Bangladesh | 746 | 95 (23.0%) | 318 (77.0%) | 413 |
| Brazil | 769 | 117 (19.6%) | 479 (80.4%) | 596 |
| Burundi | 637 | 62 (32.00%) | 132 (68.0%) | 194 |
| Chilie | 389 | 37 (13.4%) | 240 (86.6%) | 277 |
| China | 567 | 61 (16.6%) | 307 (83.4%) | 368 |
| Ecuador | 518 | 53 (21.5%) | 194 (78.5%) | 247 |
| Egypt | 1283 | 87 (36.3%) | 153 (63.8%) | 240 |
| Ethiopia | 749 | 52 (18.6%) | 227 (81.4%) | 279 |
| Greece | 597 | 133 (26.6%) | 367 (73.4%) | 500 |
| India | 495 | 41 (19.2%) | 173 (80.8%) | 214 |
| Indonesia | 1992 | 114 (15.2%) | 634 (84.8%) | 748 |
| Iraq | 336 | 71 (32.6%) | 147 (67.4%) | 218 |
| Jordan | 985 | 139 (22.5%) | 480 (77.5%) | 619 |
| Kenya | 853 | 44 (18.6%) | 192 (81.4%) | 236 |
| Lebanon | 601 | 79 (17.8%) | 364 (82.2%) | 443 |
| Libya | 876 | 54 (14.7%) | 313 (85.3%) | 367 |
| Mexico | 390 | 48 (20.3%) | 189 (79.8%) | 237 |
| Morrocco | 938 | 111 (21.4%) | 407 (78.6%) | 518 |
| Nepal | 406 | 33 (17.7%) | 154 (82.4%) | 187 |
| Pakistan | 690 | 48 (19.1%) | 203 (80.9%) | 251 |
| Palestine | 1214 | 49 (10.1%) | 435 (89.9%) | 484 |
| Qatar | 350 | 28 (12.8%) | 190 (87.2%) | 218 |
| Romania | 950 | 152 (38.5%) | 243 (61.5%) | 395 |
| Saudi Arabia | 795 | 72 (14.2%) | 437 (85.9%) | 509 |
| Sudan | 570 | 46 (28.4%) | 116 (71.6%) | 162 |
| Syria | 1500 | 57 (16.4%) | 290 (83.6%) | 347 |
| Turkey | 503 | 67 (19.0%) | 285 (81.0%) | 352 |
| United Arab Emirates | 370 | 51 (20.4%) | 199 (79.6%) | 250 |
| United States | 598 | 63 (16.3%) | 323 (83.7%) | 386 |
| Yemen | 576 | 21 (12.5%) | 147 (87.5%) | 168 |

Table S2. Univariate Logistic Regression Analysis of Long-COVID Risk Factors.

| **Variable** | **OR** | **95% CI** | **p-value** |
| --- | --- | --- | --- |
| Age | 1 | 1.00, 1.01 | 0.014 |
| Female sex | 2.08 | 1.88, 2.30 | <0.001 |
| BMI | 1.01 | 1.00, 1.02 | 0.014 |
| **Ethnicity** |  |  |  |
| White/Caucasian | Ref |  |  |
| Hispanic or Latino | 1.22 | 1.02, 1.45 | 0.028 |
| Middle eastern | 0.88 | 0.78, 0.98 | 0.026 |
| Black or African American | 0.36 | 0.28, 0.45 | <0.001 |
| Asian | 0.43 | 0.37, 0.50 | <0.001 |
| Indian | 0.51 | 0.40, 0.65 | <0.001 |
| Other | 0.76 | 0.63, 0.91 | 0.003 |
| **Smoking** |  |  |  |
| Non-Smoker | Ref |  |  |
| Current Smoker | 0.78 | 0.68, 0.89 | <0.001 |
| Ex-smoker | 0.9 | 0.72, 1.11 | 0.3 |
| Smoking duration (Years) | 0.97 | 0.94, 1.00 | 0.088 |
| Number of cigarettes | 0.99 | 0.98, 1.00 | 0.07 |
| **Respiratory parameters** |  |  |  |
| Anosmia | 0.92 | 0.82, 1.03 | 0.14 |
| Asthma | 1.1 | 0.93, 1.30 | 0.2 |
| Chest pain | 2.01 | 1.80, 2.23 | <0.001 |
| Cough | 0.96 | 0.88, 1.06 | 0.4 |
| Runny nose | 0.93 | 0.85, 1.03 | 0.2 |
| SOB | 1.83 | 1.66, 2.01 | <0.001 |
| Sore throat | 1.13 | 1.03, 1.24 | 0.009 |
| **Constitutional and Neurologic** |  |  |  |
| Confusion | 1.13 | 0.97, 1.32 | 0.12 |
| Conjunctivitis | 0.84 | 0.67, 1.05 | 0.13 |
| Dysgeusia | 1.61 | 1.46, 1.77 | <0.001 |
| Fatigue | 1.98 | 1.80, 2.18 | <0.001 |
| Fever | 1.08 | 0.99, 1.18 | 0.1 |
| History stroke | 0.97 | 0.60, 1.51 | 0.9 |
| Migraine | 1.68 | 1.48, 1.91 | <0.001 |
| Muscle joint | 1.98 | 1.80, 2.18 | <0.001 |
| Rash | 0.88 | 0.71, 1.08 | 0.2 |
| GI symptoms | 1.45 | 1.33, 1.59 | <0.001 |
| Insomnia | 1.54 | 1.32, 1.79 | <0.001 |
| **Comorbidities** |  |  |  |
| Anemia | 1.55 | 1.34, 1.80 | <0.001 |
| Autoimmune disease | 1.42 | 1.15, 1.73 | <0.001 |
| Cancer | 1.33 | 0.79, 2.15 | 0.3 |
| COPD | 1.28 | 0.84, 1.88 | 0.2 |
| Type 1 diabetes | 0.72 | 0.48, 1.05 | 0.1 |
| Type 2 diabetes | 1.04 | 0.82, 1.30 | 0.8 |
| Immunosuppression | 1.29 | 0.98, 1.69 | 0.06 |
| GI disease | 1.92 | 1.60, 2.29 | <0.001 |
| Heart disease | 0.95 | 0.73, 1.24 | 0.7 |
| Hypertension | 1.07 | 0.91, 1.25 | 0.4 |
| Renal disease | 1.21 | 0.80, 1.79 | 0.4 |
| Seizure | 0.93 | 0.59, 1.41 | 0.7 |
| **Isolation** |  |  |  |
| No Isolation | Ref |  |  |
| Home isolation | 1.46 | 1.12, 1.93 | 0.007 |
| Hospital isolation in floor | 1.03 | 0.74, 1.43 | 0.9 |
| Hospital isolation in ICU department | 2.5 | 1.71, 3.68 | <0.001 |
| Reinfection | 1.33 | 1.19, 1.48 | <0.001 |
| COVID vaccine received | 0.58 | 0.52, 0.64 | <0.001 |
| Doses | 0.88 | 0.86, 0.91 | <0.001 |

OR; Odds Ratio, CI; Confidence Interval, BMI; Body Mass Index, SOB; Shortness of Breath, GI; Gastrointestinal, COPD; Chronic Obstructive Pulmonary Disease, Ref: Reference variable.
